# Influences of parental monitoring and school connectedness on age at first sexual debut among unmarried female youth in Bedele town, Ethiopia: A survival analysis of timing using accelerated failure time model

**DOI:** 10.1371/journal.pone.0271906

**Published:** 2022-07-26

**Authors:** Gebiso Roba Debele, Dereje Tsegaye, Teshale Gemechu, Sabit Zenu Siraj

**Affiliations:** 1 Department of Public Health, College of Health Sciences, Mettu University, Mettu, Ethiopia; 2 Bedele General Hospital, Bedele, Ethiopia; Flinders University, AUSTRALIA

## Abstract

**Background:**

The age of first sexual debut among youth continues to attract interest as it marks the start of their exposure to a variety of sexual and reproductive health problems. Parental monitoring (PM) and school connectedness (SC) has been found to have an effect on this problem. Despite this fact, there is a dearth of literature on implications of PM and SC on age at first sexual debut in Ethiopia. Therefore, this paper examined the influences of PM and SC age at first sexual debut among female youth.

**Methods:**

A retrospective follow-up study was conducted on 470 randomly selected female youth in Bedele town, Oromia regional state, southwest Ethiopia from February 1 to March 30, 2021. The age at first sexual debut was asked in full years for respondents who ever engaged in sexual debut at a time of data collection. Data were entered using Epi-Data version 4.6 and analyzed using Stata version 14. All variables at P-values less than 0.2 in bivariable analysis were exported to multivariable analysis. Multivariable Accelerated Failure Time (AFT) regression analyses using Weibull distribution were used to examine the association of age at first sexual debut with PM and SC at 5% level of significance.

**Results:**

Overall, 132(28.1%) youths were begun sexual activities of which 79.54% was an early (vaginal penetrative sex before 18 years old). The incidence rate was 15.58/1000 PY with 95% CI of [13.14, 18.47]. The result shows that, female youth start first sexual debut as early as 10 year and mean age was 16.89±2.82. Only 27.97% of those who began sexual debut used contraception during first sex. Multivariable Weibull AFT regression analyses adjusted for different variables showed that high PM (Adjusted Time Ratio (ATR) 1.13: 95%CI; [1.04, 1.21]) and good SC (ATR: 1.14: 95% CI; [1.06, 1.22] significantly delays the age at first sexual debut.

**Conclusions:**

Overall, four out of every five premarital sexual activities were early sexual debuts. High PM and high SC significantly decrease early sexual debut by delaying the age of sexual initiation. Therefore, family and school involvement focused on PM and SC of the youth is recommended as an important mechanism for preventing youths’ risky sexual behaviour, including early sexual debut.

## Introduction

Youth (aged 15–24 year) is thought to be a very challenging period of time when many significant life events occur **[1]**. There were over 1.2 billion youth in 2019 representing 16% of global population with the peak in Sub Saharan Africa (SSA) next to Asia **[2]**. Surprisingly, about 30% of Ethiopian population are youth according to 2018 United States Agency for International Development (USAID) report **[3]**. The age of first sexual debut among youth continues to fascinate much interest because it marks the start of their exposure to a variety of Sexual and Reproductive Health (SRH) problems **[4, 5]**. Despite a decline of some adolescent sexual risk behaviors in the last decade, a decrease in the age at sexual initiation have occurred during the past 30 years **[6, 7]**.

Sexuality is a normal part of healthy youths adaptable developmental milestone **[8]**. Early initiation, on the other hand, is linked to later SRH problems, such as increased incidence of Sexually Transmitted Infections (STIs) **[9]**, unintended pregnancy **[10]**, early pregnancy and sex with risky partners **[11]**. In Ethiopia, the median age at first sexual intercourse was 16.6 for female, which was 0.5 years younger than the median age of first marriage **[12]**. This indicates women engage in sex before marriage. More than half (66.2%) of those who had their first sexual intercourse was early sexual debut according to 2016 Ethiopian Demographic and Health Survey (EDHS) **[13].**

Africa is the home to the largest number of Human Immunodeficiency Virus (HIV) infections (20.6 million) with peak in SSA **[14]**. Of an estimated daily 6000 new infections that occur globally, about two-thirds are in SSA **[15]**. In SSA countries 80% of all HIV infections occurring among youth occur among youth girls aged 15–19 years **[16]**. With a prevalence of 1.1% and an incidence of 0.33/1000 people, HIV infection is a major public health concern in Ethiopia **[17]**. Therefore, policies aimed at protecting young people from new infections through behavioral change programs is required in SSA countries including Ethiopia **[18]**. Of those behavioral change domains, early sexual debut prevention needs to be the main target.

Despite of the increasing proportion of youth engaging in early sexual initiation, contraceptive use remains low in SSA **[19]**. For example, only 50.2% of age 20–24 and 36.5% of age 15 to 19 females use modern contraception in Ethiopia according to 2019 mini EDHS **[20]**. In Ethiopia, unintended pregnancy and teenage pregnancy is about 26.6% **[21]** and 16%, respectively **[12]**.

Poverty provokes youth especially females into sexual relationships to help themselves and their families **[22]**. Beyond poverty, the age at first sexual debut is profoundly influenced by personal and social circumstances and, particularly, parents and school environment **[23]**. Youth behaviour particularly when it relates to the parents is paramount to social work as a discipline because of the broad implications of such behaviour on the family and society at large **[24]**. Social work helps youths develop problem-solving skills through a multidisciplinary approach, which reduces risky sexual behavior **[25].** Youths SRH is not only central to the immediate health outcome but also has consequences on shaping future adult health **[26]**. For youth, among the most influential of these circumstances is the social factors like family in particular, parents and school **[27, 28]**.

Family’s social characteristics such as PM and SC have important implication on the behaviour of children and their achievements throughout their life course **[29]**. Evidence has jagged to the influence of family on young person’s sexual behaviour **[30]**. Supportive parental ties, for example, have been shown to provide children more confidence in their ability to face academic obstacles and avoid negative peer influences. **[31]**. Youth whose Parents monitor well can influence their parents’ behaviour later and play a role in generating the very social conditions that influence adverse developmental outcomes **[32]**.

Because previous studies have shown the role of parents and school in teenage sexual behavior, such interventions have become more common. **[33, 34]**. Parenting through monitoring has been found to have an effect on young people’s sexual behaviour **[35]**. Evidence from different western countries supports the positive impact of parents and school on the SRH of youth **[36, 37]**. Parents can modify sexual decision-making among youth by PM and connecting their child to the school, so that early sexual debut decreases **[38]**. SC is crucial for students’ health because they are less likely to engage in risky sexual behavior when they feel connected to their school **[39]**.

Several studies have demonstrated a link between young people’s risky sexual behavior and levels of PM, and SC in western countries. However, significantly less research has focused on the influences of PM and SC on age at first sexual debut in Ethiopia. Furthermore, despite the fact that early sexual debut has a disproportionate effect on females SRH across the life course compared to boys, several research include both sexes as their study subjects. In addition, the current study used advanced statistical analysis (Accelerated Failure Time (AFT) model) which used to assess whether PM and SC delays or shorten age at first sexual debut. In this regard, this study is timely and of great importance to the advancement of evidence related to PM and SC influence on age at sexual initiation. Thus, this paper examined the influences of PM and SC on age at sexual debut of youth female’s in Bedele town, Ethiopia using AFT model.

## Methods and materials

### Study setting, design and period

School based retrospective follow up study was conducted in Bedele town from February 1 to March 30, 2021. Bedele town is a capital city of Buno Bedele Zone located in Oromia Regional sate, at a distance of 408KM south west of Addis Ababa (capital city of Ethiopia). Buno Bedele zone is bordered on the south by the Southern Nations, Nationalities, and Peoples Region, on the west by the Ilu Aba Bora Zone, on the north by the East and West Wollega Zones, and on the east by the Jimma Zone. In Ethiopia, high school education includes the students from ninth to the twelfth grade. According to data adopted from urban planning institute Bedele town strategic plan in 2020, the estimation of female youth among the population was 5099. Bedele town has two high schools and one college Dabena Poly Technique College (DPTC). Woyessa Gota high school is one of the schools located on the north direction of Bedele town in Nekemte roadway and Ingibi high school is also located southwest of Bedele town on the way of Jimma town. DPTC is located on the way to Mettu town road in the western direction nearer to high way next to Bedele General Hospital.

### Study population and sampling

The study considered all regular female youth in the two high schools and DPTC who were available during data collection period. Those students with severe medical or mental illness that necessitates missing classes during the course of study were excluded from the study. Sample size for this study (489) was determined using factors significantly associated with time to first sexual debut from previous studies using Schoenfeld formula **[40]** under Stata software version 14.

**Fig 1:**
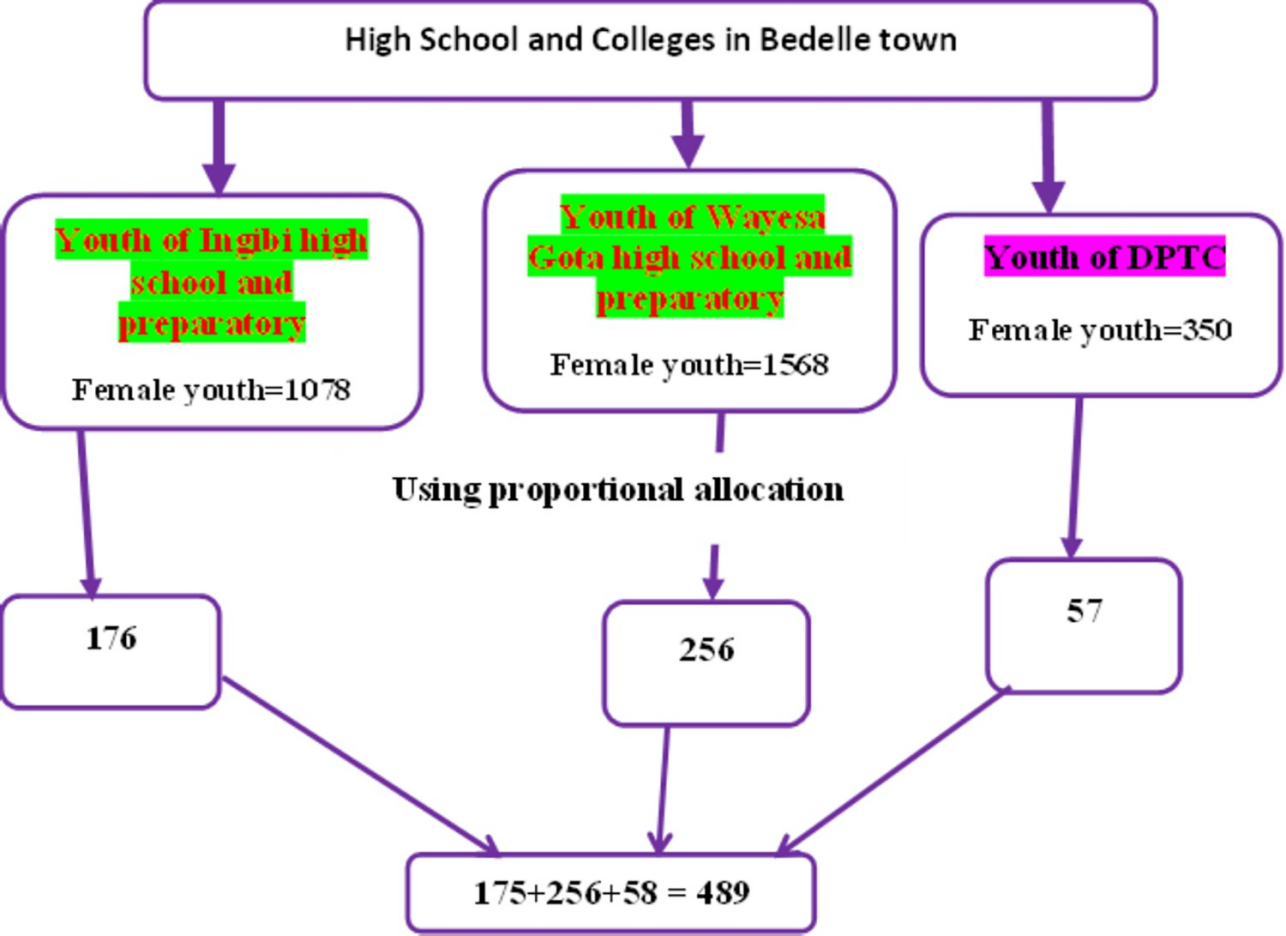
Schematic presentation of the sampling procedure used in the study, 2021.

The total number of female youth students in Woyessa Gota high school, Ingibi high school, and DPTC are 1568, 1078 and 350 respectively and adopted from their respective institutions. The list of students was available in the school registrar and thus, the students’ ID number was used as sampling frame. Then, simple random sampling techniques using a random number table was applied after proportional allocation was done to both high schools and DPTC. The schematic presentation of the sampling procedure was summarized in **Fig 1.**

### Study variables and measurements

The primary outcome of interest was time to first sexual debut and this outcome was derived from two questions in the instruments. The first question asked the respondents was whether they had ever engaged in sexual intercourse. The second question asked was, the age (in full years) at first sexual intercourse to get the events time. The youth that had never engaged in sexual intercourse at a time of data collection was considered as censoring. The focal independent variable in this study were PM and SC measured using standard tool which operationalized below and their association with age at first sexual debut was examined using AFT model of parametric survival analysis after adjusting for confounding variables.

#### Youth

According to United Nation (UN), youth is defined as an individual aged 15 to 24 years **[41]**.

#### Sexual debut/intercourse

All sex performances that are penetrative penile to Vagina and considered early if they experience before 18 years of age **[13, 42, 43]**.

#### Time to first sexual debut

The time (in years) between birth and event (first sexual intercourse).

#### Parental monitoring

The family and the other significant relatives of female youth monitoring while they are out of home for some reason. It was assessed using a six-item Silverberg’s parental monitoring scale **[44]**. The scale assesses the perceptions of whether the parent(s) usually aware of their youth’s activities and whereabout. The scale of measurement include: (1) My parents know where I am after school, (2) If I am going to be home later, I am expected to call my parents to let them know, (3) I tell my parents who I am going to be with before I go out, (4) When I go out at night, my parents know where I am, (5) I talk with my parents about the plans I have with my friends, and (6) When I go out, my parents ask me where I am going. All Items were scored from 1 (never) to 5 (always) and those who scored lower than the median value was considered as low PM and good PM for those who scored above the median **[45]**.

#### School connectedness

The five-item Likert scale that adopted and developed from a previous study was used to assess school connectedness **[46]**. The scale measurement includes; 1) do you feel close to people who are at school 2) do you feel happy to be at this school 3) do you feel as if you are a part of this school 4) do school teachers treat students fairly at this school 5) do you feel safe being at this school. The responses of each item ranged from ‘strongly disagree’ (coded 1) to ‘strongly agree’ (coded as 5). The scale was classified into poor and good SC after principal component analysis using quantiles **[43]**.

#### Attitude towards STIs

The 8 items five-level Likert scale that were arranged from strongly disagree to strongly agree was used to assess attitude towards STIs **[47]**. Female youth who scored greater or equal to median were considered as having favorable attitude towards STIs **[47, 48]**.

#### Knowledge about STIs

Was assessed using the information difference of youth about the mode of transmission, sign and symptoms and way of prevention along with control of STIs. each correct answer was coded 1 and wrong answer was scored 0. Youth students who scored above the mean score were considered as having good knowledge **[48, 49]**.

### Data collection and quality assurance

A structured self-administered questionnaire, which was adapted after a thorough review of literature, was used to collect data. The questionnaire comprehends of socio-demographic characteristics of youths and parents (age, education level, parental education, parental occupation, sexual issue discussion with their parents, religious participation), PM, SC, knowledge and attitude towards STIs. The data collection tool was prepared in English, translated into Afan Oromo, and then back to English to check its consistency. One BSc nurse was assigned as supervisor and three female BSc nurse in collaboration with three female teachers were assigned as a facilitator data collection.

To ensure the quality of the data, two-day training was given for of the data collectors and supervisors by the principal investigator on the objectives, relevance of the study, methods of responding to the self-administered questionnaire, the confidentiality of the information, and informed consent. A pre-test was done on 5% of the study participants out of Bedele town before the actual data collection work to see for the accuracy of responses and to estimate the time needed and the questionnaire was adjusted accordingly. Before data collection, the trained data collectors were giving orientation to female youths regarding the aim of the study, the content of the questionnaire, the issue of confidentiality, and respondents’ rights. Moreover, trained data collectors were involved in collecting consents from participants and gathering filled questionnaires.

### Statistical analysis

The data were checked for inconsistencies, coding, completeness, and clarity before entry. The data entry was done using Epi-Data version 4.6 and then exported to Stata version 14 for further data cleaning and analysis. Descriptive measures such as means, percentages, frequencies, and standard deviations were used to characterize the study population.

Time to first sexual debut was estimated using Kaplan-Meier (KM) method and the Log-rank test was used to compare survival time between groups of categorical variables. A significant variable at p values less than 0.2 in the bivariate analysis was included in the multivariable survival models. The multicollinearity of the variables was assessed using pseudo–Variance Inflation Factor (VIF). The best-fitting survival model was selected based on Akaike information criteria (AIC). Then multivariable using the best-fitted survival model was analyzed using Stata. Cox Snell residual plot was used for checking final model adequacy. Finally, multivariable AFT regression survival analyses using Weibull distribution were used to examine the influences of PM and SC on age at first sexual debut at 5% level of significance.

## Result

### Socio demographic characteristics of respondents

Of 489 samples, 470 were participated in the study yielding 96.11% response rate. Nearly two-thirds 304 (64.68%) of female youth found in the age group of 15–19 years and majority 292 (62.13%), of them were urban residents. Sixty-five (13.83%) of fathers and 106 (22.55%) mothers of the female youth had no formal education. Most 440 (93.6%), of the students had a religious participation and more than half 259 (55.11%) of them participate once a week **Table 1**.

**Table 1:** Socio-demographic characteristics of the respondents among female youths of Bedele town, 2021 (n = 470).

Variable	Category	Frequency	Percentage
Age	15–19	304	64.68
	20–24	166	35.32
Educational status of students	High school	247	52.6
	Preparatory	172	36.6
	DPTC	51	10.9
Residence	Rural	178	37.87
	Urban	292	62.13
Religion	Orthodox	172	36.6
	Protestant	174	37.0
	Muslim	124	26.4
Religious Participation	Yes	440	93.6
	No	30	6.4%
Frequency of religious participation	Every day	44	9.36
	Once a week	259	55.11
	Once a month	153	32.55
	Once a year	14	2.98
Person with living	Parents	305	64.89
	Alone	34	7.23
	Relative	116	24.68
	Grandparents	15	3.19
Educational status of the father	No formal education	65	13.83
	primary (1–8 grade)	144	30.64
	Secondary (9–12 grade)	146	31.06
	Diploma and above	115	24.47
Educational status of the mother	No formal education	106	22.55
	primary (1–8 grade)	188	40.00
	Secondary (9–12 grade)	112	23.83
	Diploma and above	64	13.62
Father’s employment status	Civil servant	115	24.68
	Private employer	79	16.95
	Merchant	117	25.11
	Daily laborer	17	3.65
	Farmer	125	26.82
	Others	13	2.79
Mother’s employment status	Housewife	162	34.62%
	Civil servant	78	16.67%
	Private employer	62	13.25%
	Merchant	77	16.45%
	Daily laborer	11	2.35%
	Farmer	78	16.67%

### Substance and psychosocial factors

Twenty-eight (5.94%) and 20 (4.26%) of the study participants reported drinking alcohol and chewing khat respectively. Nearly one-fifth (18.28%) had a peer pressure to begin sexual debut. About 52.12% of the study participants had good knowledge towards STIs and 53.19% of participants had a favorable attitude towards STIs. Moreover, nearly two-thirds (62.7%) of students had a communication about sexuality and RH with their families **Table 2.**

**Table 2:** Substance and Psychosocial factors characteristics of the study participants.

Variables	Category	Frequency	Percentage
Ever used alcohol	Yes	28	5.94%
	No	442	94.04%
Ever used Khat	Yes	20	4.26%
	No	420	95.74%
Ever encountered peer pressure	Yes	88	18.28%
	No	382	81.28%
Relationship with partner at fist sexual intercourse	Fiancé	18	13.64%
	Boyfriend	116	86.36%
Have Boyfriend	No	392	83.40%
	Yes	78	16.60%
Watching Pornography	No	368	78.30%
	Yes	102	21.70%
Ever discussed with your parents about SRH?	No	175	37.23%
	Yes	295	62.77%
Knowledge towards STIs	Poor knowledge	245	52.13%
	Good knowledge	225	47.87%
Attitude towards STIs	Favorable	250	53.19%
	Unfavorable	220	46.81%

Abbreviation: STIs; Sexually Transmitted Infections, SRH; Sexual and Reproductive Health

**Fig 2:**
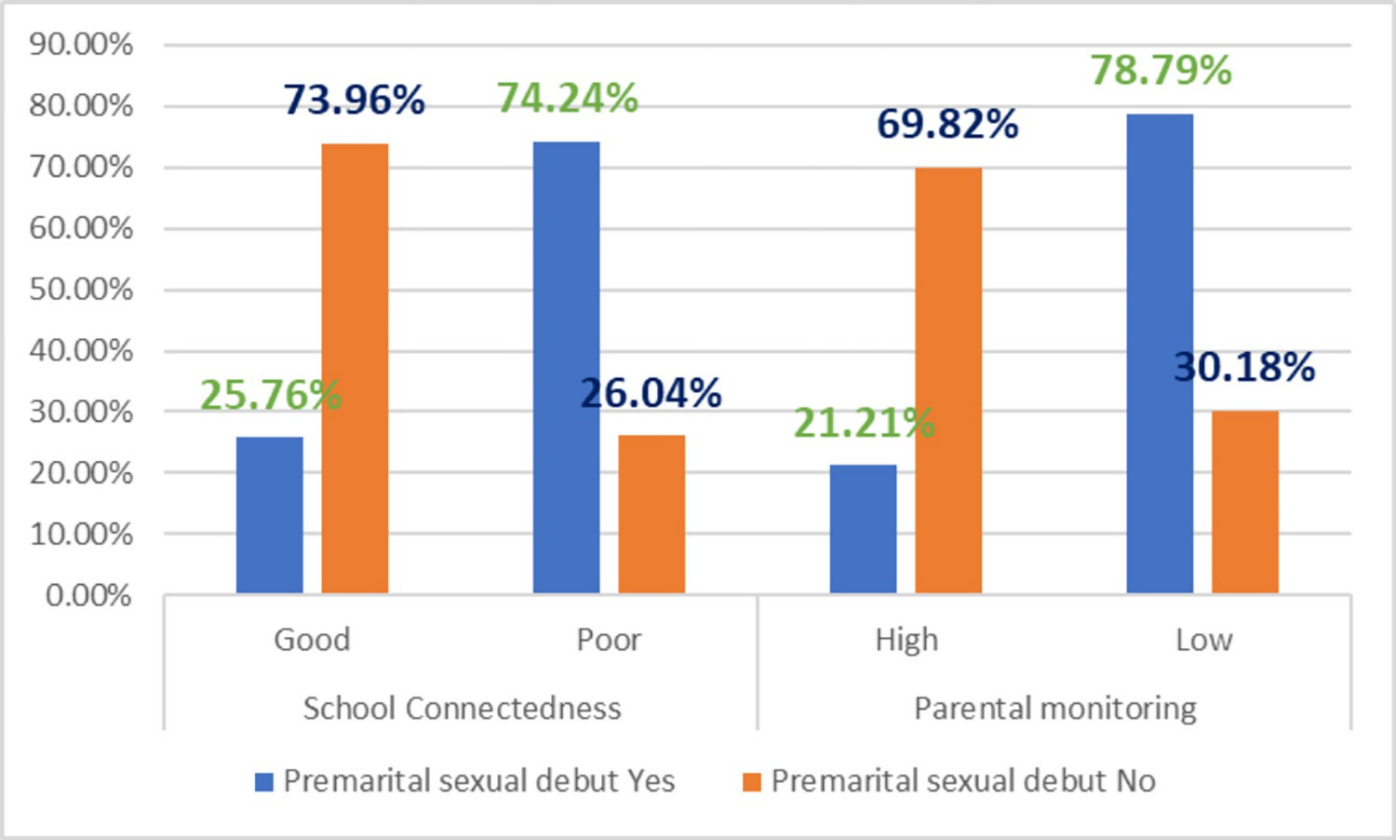
First premarital sexual debut variation on school connectedness and parental monitoring among female youth in Bedele town, Ethiopia.

**Fig 3:**
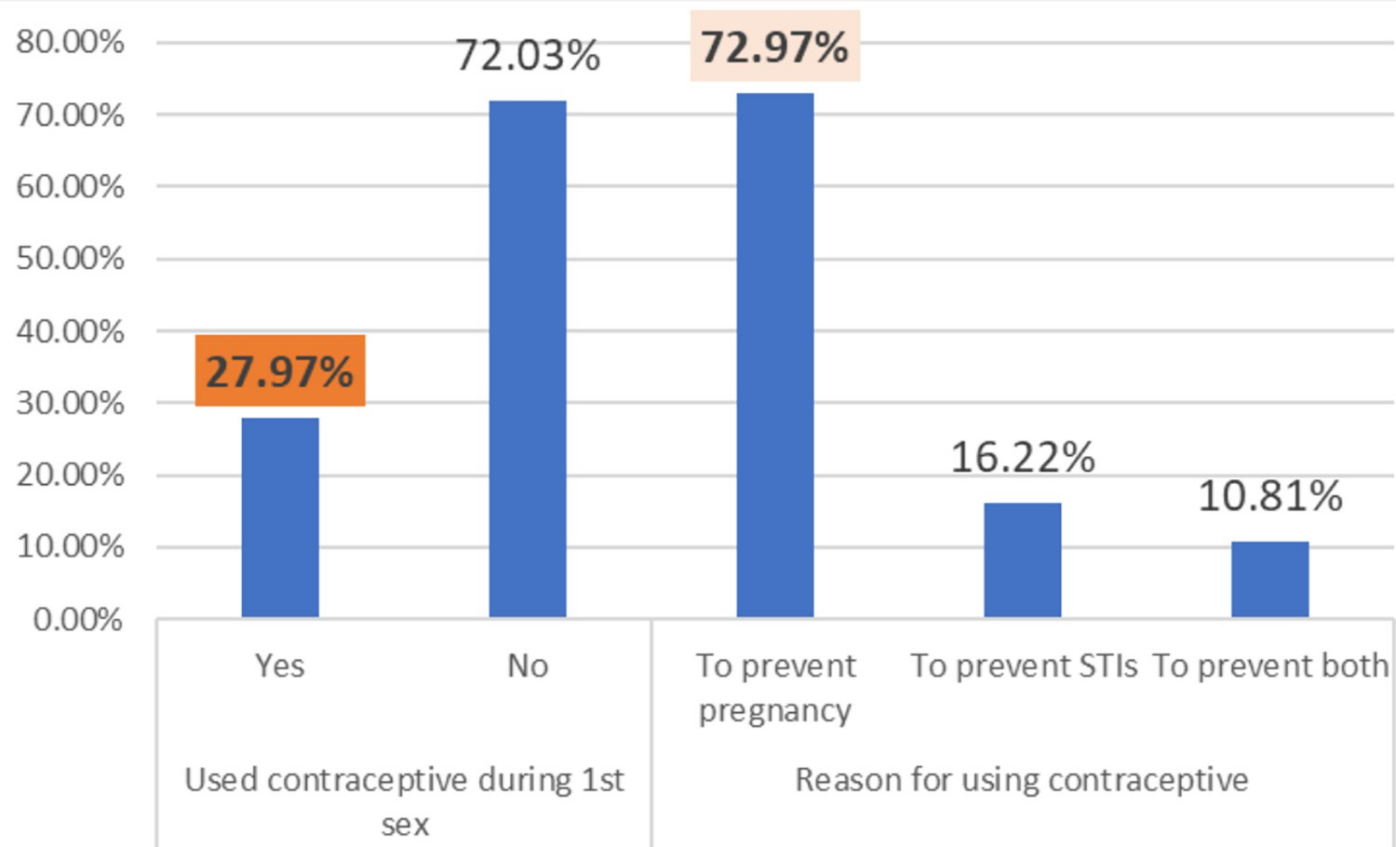
Contraceptive usage of female youth during their first sexual debut in Bedele town, Ethiopia.

Premarital sexual debut is higher among those students with poor SC and low PM compared to their counter parts **(Fig 2).** Only 27.97% who made their sexual debut took contraceptives at their first encounter, while the majority (72.9%) utilized contraception to avoid pregnancy **(Fig 3).**

### Incidence of premarital and early sexual debut

**Fig 4:**
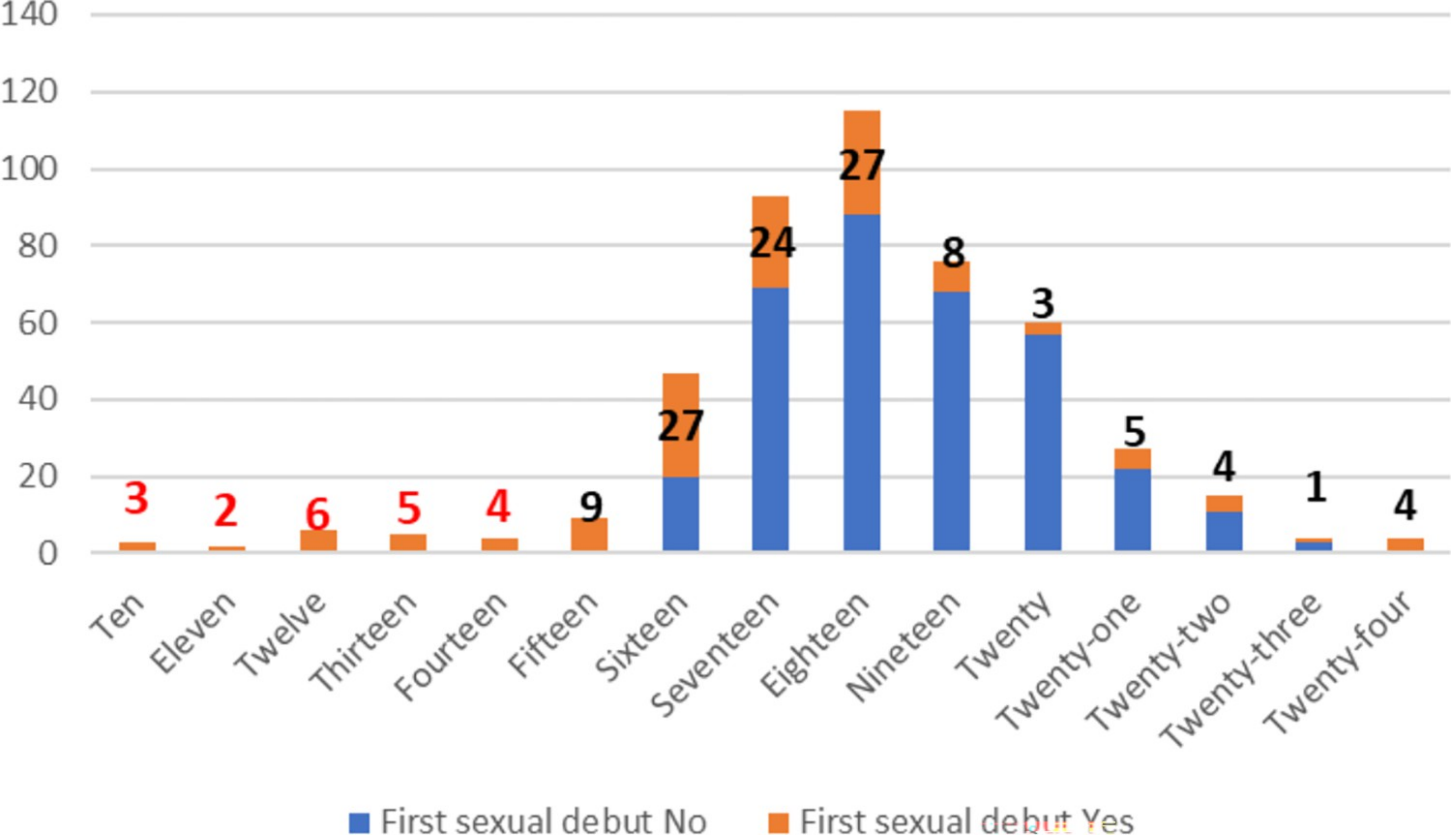
First sexual debut at each age of female youth in Bedele town.

**Fig 5:**
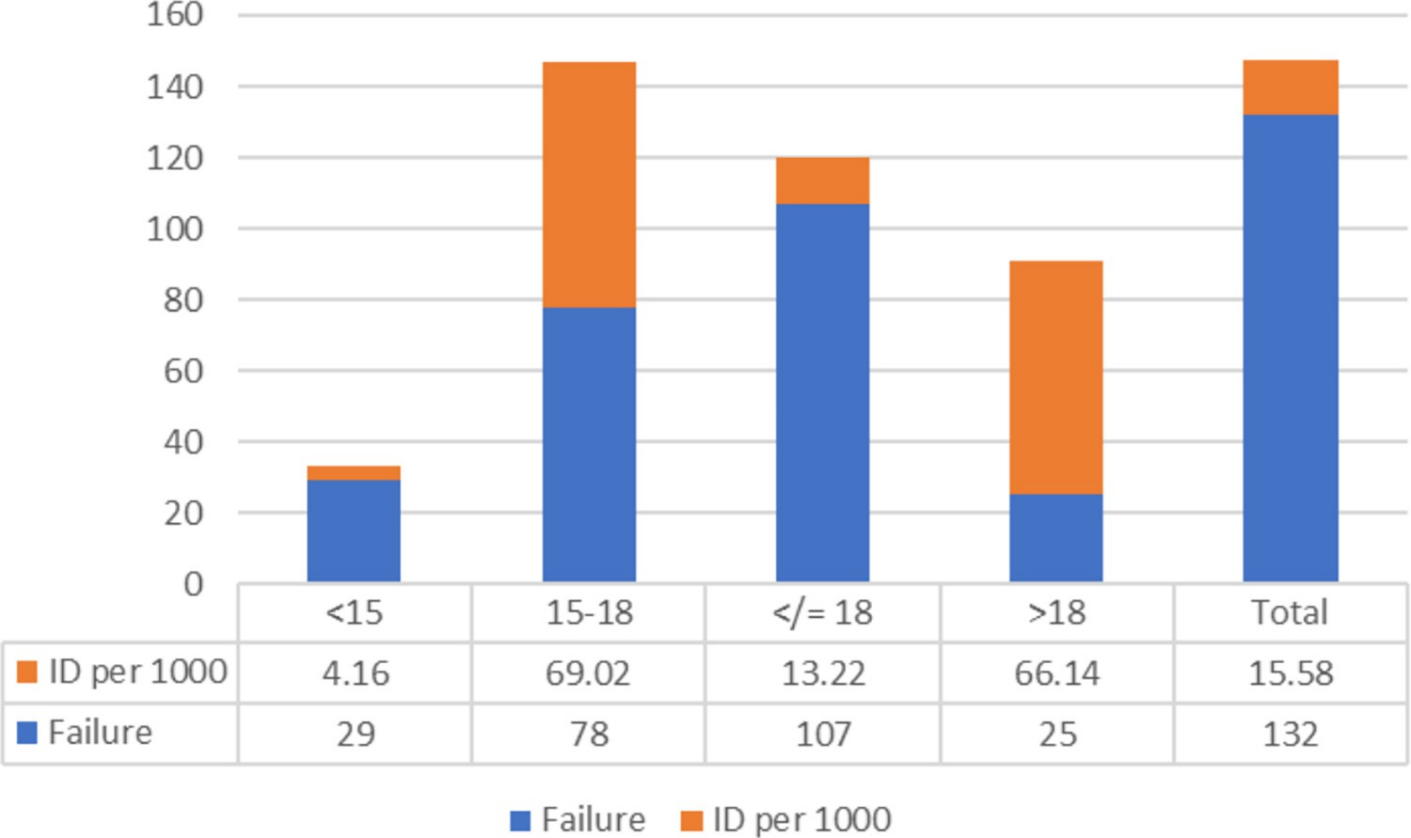
Incidence rate of first sexual debut among female youth of Bedele town with their respective number of failures.

At the end of the follow-up time, a total of 132(28.1%) female youth were begun first sexual intercourse of which 79.54% was an early sexual debut. This indicates that almost four in five of premarital first sexual debut is early. The result shows that, female youth start first sexual debut as early as 10 years with the mean age of 16.89 ±2.82 **(Fig 4)**. The overall incidence density (ID) of premarital sexual debut was 15.58 per 1000-person year (PY) with 95% CI of [13.14, 18.47] after 8473 PY observation. The ID of early sexual debut was 13.22 per 1000 PY, 95% CI (10.94, 15.98) and twenty-nine female youth started sexual debut before 15 years with ID of 4.16 per 1000 PY. The ID was reported with respective number of failures by the time interval **(Fig 5)**.

### Survival probability of the study participants

**Fig 6:**
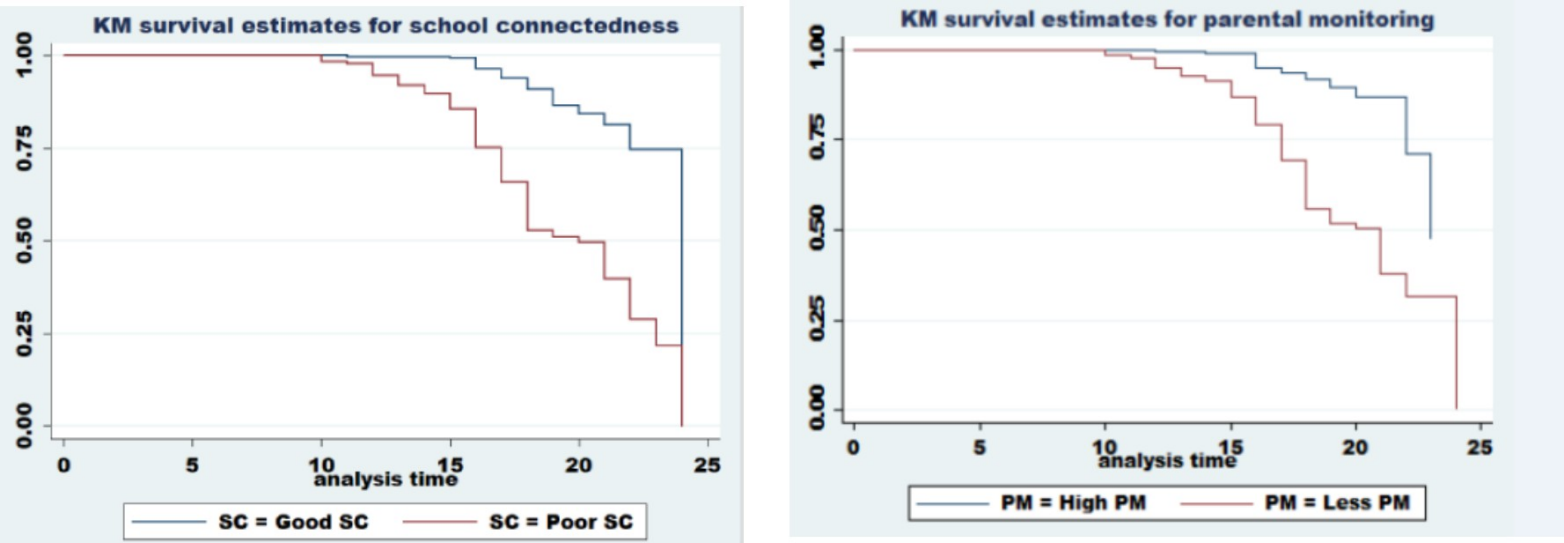
Kaplan Meier survival estimates for different categories of parental monitoring and school connectedness among female youth in Bedele town.

A separate graph of KM survival estimates had been constructed for both variables to see the existence of differences in survival estimates. Survival probability was higher among patients who had high PM and good SC compared to their counter categories **(Fig 6)**. Based on the result of the log-rank test there was a significant difference in survival among those categories.

### Influences of SC and PM on age at first sexual debut

**Fig 7:**
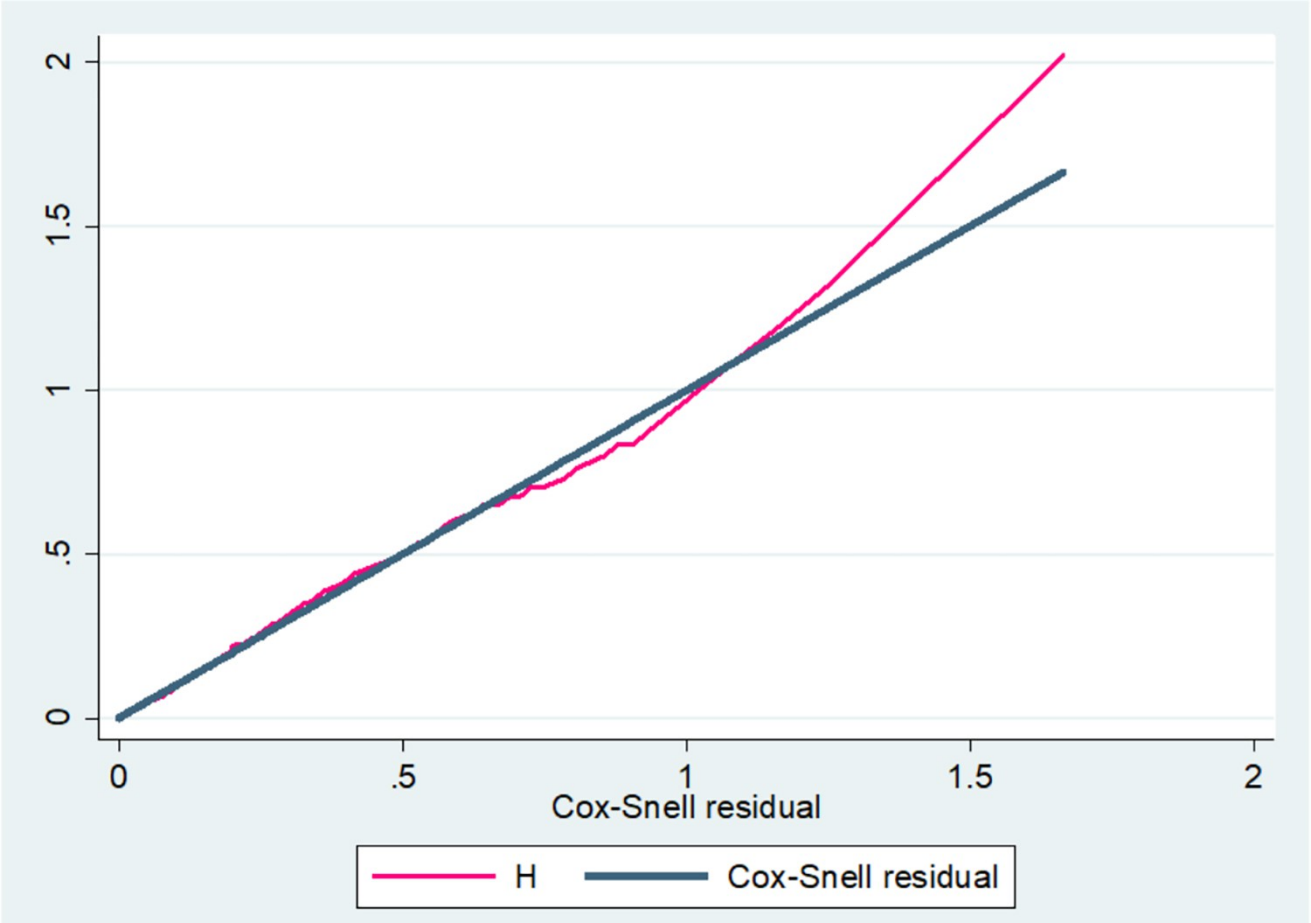
Final model adequacy graph based on cox snell residual plot.

First and foremost, we used Cronbach’s alpha to assess the internal consistency of the SC and PM tools. A result of Cronbach’s alpha for PM and SC was 0.7761 and 0.7612, respectively. The items are internally consistent to measure the intended variable because all values are greater than 0.7. As variable selection precedes model diagnostics, factors significantly associated with time to sexual debut in the bivariate analysis at p values less than 0.2 were included in the multivariable survival model. The multicollinearity of the variables was assessed using pseudo VIF and its values range from 1.04 to 1.61 which indicates the absence of multicollinearity among the independent variables. The AFT model with Weibull baseline distribution was selected as a parsimonious model compared to other because of lowest AIC (147.08). The goodness of fitness of the model was satisfied after checked by using Cox Snell residual pot **(Fig 7)**. A multivariable Weibull AFT model shows as good SC [ATR = 1.14; 95% CI (1.06, 1.22)] and high PM [ATR = 1.13; 95% CI (1.04, 1.21)] delays age at first sexual debut among female youth independent of residence, maternal education, parent youth discussion about SRH, peer pressure, exposure to pornographic material, knowledge and attitude towards STIs **Table 3.**

**Table 3:** Multivariable AFT regression analysis model with Weibull distribution.

Variable	Category	Survival status		CTR with 95% CI	ATR With 95% CI
		Event	Censored		
Residence	Urban	62	230	1	1
	Rural	70	108	0.91(0.86, 0.96)	0.97 (0.91, 1.03)
Mothers’ educational status	Diploma and above	11	53	1	
	No formal education	40	66	0.89 (0.80, 0.99)	1.02 (0.91, 1.15)
	Primary	61	127	0.93 (0.84, 1.03)	1.07 (0.96, 1.19)
	Secondary	20	92	1.02 (0.91, 1.14)	1.04 (0.93, 1.17)
Discussion about SRH with parents	No	44	131	1	1
	Yes	88	207	0.98 (0.92, 1.03)	1.04 (0.98, 1.10)
Parental Monitoring	Low	104	102	1	1
	High	28	236	1.29 (1.20, 1.39)	1.13 (1.04, 1.21***
Peer pressure	No	61	321	1	1
	Yes	71	17	0.75 (0.70, 0.80)	0.85 (0.79, 0.92)
Having Boyfriend with history of sexual intercourse	No	72	320		
	Yes	60	18	0.79 (0.74, 0.84)	0.97 (0.90, 1.04)
Exposure to pornographic material	No	61	307	1	1
	Yes	71	31	0.78 (0.74, 0.84)	0.95 (0.88, 1.02)
School Connectedness	Poor	98	88	1	1
	Good	34	250	1.28 (1.19, 1.37)	1.14 (1.06, 1.22) **
Attitude towards STIs	Favorable	34	216	1	1
	Unfavorable	98	122	0.83 (0.78, 0.89)	0.93 (0.87, 1.00)
Knowledge towards STIs	Good knowledge	52	173	1	1
	Poor knowledge	80	165	0.95(0.90, 1.00)	0.96 (0.91, 1.02)

Abbreviation: ATR; Adjusted Time Ratio, CTR; Crude Time Ratio, AFT; Accelerated Time Ratio, STIs; Sexually Transmitted Infections, SRH; Sexual and Reproductive Health

*** p < 0.001

** p < 0.01

## Discussion

In this study, we enrolled 470 participants and analyzed the association of PM and SC with age at first sexual intercourse. In this study, 132 youths (28.1%) started premarital sexual debut, with 79.54% starting early. This indicates almost 4 out of every 5 of premarital sexual debut was early. The ID premarital sex was 15.58 per1000 PY with 95% CI of [13.14, 18.47]. The ID of early sexual debut was 13.22 per 1000 PY, 95% CI (10.94, 15.98). The result shows that, female youth start first sexual debut as early as 10 years with mean age of 16.89 ±2.82. Multivariable Weibull AFT model adjusted for different variables showed that, high PM and good SC significantly delays the age at first sexual debut.

In this study, we hypothesized that a good perceived PM may effort to delay age at first sexual onset by providing the opportunities for youths to act on their own intention to engage in sexual behavior. The current study found as successful high PM to be significantly delays age at first sexual debut even after accounting for the influences of the other important determinants **(Table 3)**. This suggests that parental supervision and control on youths affects their tendency of being involved in sex. This result is in tandem with previous studies **[43, 50–52]**, which confirmed a significant relationship between PM with youths not having sexual intercourse till a later date. This is an important finding because it emphasizes the necessity of PM efforts in preventing early sexual intercourse in order to reduce additional reproductive consequences. Family can modify sexual decision-making among youth by parenting through monitoring so that early sexual debut decreases **[53]**. When parents make a habit of knowing about their teens (what they are doing, who they are with where they are and setting clear expectations for behavior with regular check-ins), they can reduce their teens’ risks sexual behaviour **[54]**. Other study shows that teens whose parents use effective monitoring practices are less likely to make poor decisions, like having sex at an early age **[55]**.

This could be because youths who believe they are successfully monitored by their parents are more likely to consider their parents’ sexual behavior and social expectations when determining their own likelihood of engaging in intercourse. This reasoning is supported by different other studies **[56, 57]**, associating youth perceptions of parental disapproval with the early onset of sexual debut. UN takes a view of SRH, as one of the most crucial, parts of the Sustainable Development Goals (SDGs) agenda **[58]**. The finding of the current study may contribute a little for the achievement of this goals.

A good SC can also help to prevent early sexual debut by delaying the age of sexual debut. This finding provides additional empirical evidence of the impact of SC on age at first sexual debut and has important implications from the perspective of child development. This finding also encourages the school- based interventions of SRH in delaying sexual debut among adolescents which is being implemented in some countries **[59, 60]**. This finding is supported by previous studies conducted within high- income countries **[61–63]** which showed as school- based interventions or SC was effective in delaying age at sexual debut. Similarly, a result of studies in low and middle- income countries found that good SC accelerates the age at sexual initiation **[64–66].** School is an institution where most youth spend majority of their time, for learning and interacting with peers. This could be because engaging youths with their school environment encourages them to engage in healthy behaviors and reduces risky sexual behaviors through critical thinking skills learned in schools **[59]**. This might be because messages conveyed by teachers regarding the importance of staying in school and affects the student attitudes regarding the desirability of early sexual activity. Currently, the global health community is ever more committed to adolescent SRH for improving health of young people to meet the SDGs **[67]**. Other studies found no significant effects of school- connectedness on decelerating or delaying sexual debut **[68–70].** This variation could be owing to the study population and study area being different. In that instance, our study only included unmarried female youths, whereas earlier investigations included both male and female subjects, as well as married youth.

### Strength and limitation of the study

The current study’s strength is the implementation of a sophisticated statistical model and a longer follow-up time. As per our knowledge this is the first study to assess the influences of PM and SC on age at first sexual debut using AFT model. Despite its strength, this study has some limitation. The study topic by itself assesses sensitive issues related to sexuality which might be the reason for underreporting some behaviors like incidence of premarital sexual intercourse. Due to the retrospective nature of this study, the age of sexual debut may be affected by recall bias. Outcome and exposure measurement regarding reproductive health consequences were based on self-report making our study prone to social desirability bias.

## Conclusion

Early sexual debuts accounted for four out of every five premarital sexual activities. This indicates early sexual debut remains the SRH problem in Ethiopia. Our result shows high PM and good SC significantly delays the age at first sexual debut or in word it decreases early sexual debut. Therefore, family and school involvements focused on PM and SC of the students is an important mechanism for preventing youths’ risky sexual behaviour including early sexual initiation.

## Supporting information

S1 Data This is the data set of a study of influences of parental monitoring and school connectedness on age at first sexual debut among unmarried female youth in Bedele Town, Ethiopia: A survival analysis of timing using accelerated failure time model. (SAV)

## Abbreviations

AIC: Akaike information criteria
ATR: Adjusted Time Ratio
AFT: Accelerated Failure Time
DPTC: Dabena Poly Technique College
EDHS: Ethiopian Demographic and Health Survey
ID: Incidence Density
HIV: Human Immunodeficiency Virus
KM: Kaplan-Meier
PM: Parental Monitoring
PY: Person Year
SC: School Connectedness
SDG: Sustainable Development Goal
SRH: Sexual and Reproductive Health
SSA: Sub Saharan Africa
STI: Sexually Transmitted Infection
UN: United Nation
USAID: United States Agency for International Development
VIF: Variance Inflation Factor
WHO: World Health Organization
